# ARAM: A Technology Acceptance Model to Ascertain the Behavioural Intention to Use Augmented Reality

**DOI:** 10.3390/jimaging9030073

**Published:** 2023-03-21

**Authors:** Anabela Marto, Alexandrino Gonçalves, Miguel Melo, Maximino Bessa, Rui Silva

**Affiliations:** 1 Computer Science and Communication Research Centre (CIIC), School of Technology and Management (ESTG), Polytechnic of Leiria, 2411-901 Leiria, Portugal; 2 Institute for Systems and Computer Engineering, Technology and Science (INESC TEC), 4200-465 Porto, Portugal; 3 Department of Engineering, University of Trás-os-Montes and Alto Douro, 5000-801 Vila Real, Portugal

**Keywords:** mobile augmented reality, acceptance of technology model, cultural heritage, UTAUT

## Abstract

The expansion of augmented reality across society, its availability in mobile platforms and the novelty character it embodies by appearing in a growing number of areas, have raised new questions related to people’s predisposition to use this technology in their daily life. Acceptance models, which have been updated following technological breakthroughs and society changes, are known to be great tools for predicting the intention to use a new technological system. This paper proposes a new acceptance model aiming to ascertain the intention to use augmented reality technology in heritage sites—the Augmented Reality Acceptance Model (ARAM). ARAM relies on the use of the Unified Theory of Acceptance and Use of Technology model (UTAUT) model’s constructs, namely performance expectancy, effort expectancy, social influence, and facilitating conditions, to which the new and adapted constructs of trust expectancy, technological innovation, computer anxiety and hedonic motivation are added. This model was validated with data gathered from 528 participants. Results confirm ARAM as a reliable tool to determine the acceptance of augmented reality technology for usage in cultural heritage sites. The direct impact of performance expectancy, facilitating conditions and hedonic motivation is validated as having a positive influence on behavioural intention. Trust expectancy and technological innovation are demonstrated to have a positive influence on performance expectancy whereas hedonic motivation is negatively influenced by effort expectancy and by computer anxiety. The research, thus, supports ARAM as a suitable model to ascertain the behavioural intention to use augmented reality in new areas of activity.

## 1. Introduction

Due to the technological developments of the last few decades, people now have the opportunity to access technology almost everywhere through the use of handy devices such as smartphones or tablets. Accordingly, some technological solutions, inter alia, Augmented Reality (AR), can be experienced almost everywhere. As AR becomes more popular and easier to develop, it is noticeable that there is a growing number of applications in different areas demonstrating this technology as an attractive solution. However, similarly to any new technology, and even though its advantages and benefits have been demonstrated in the literature in a variety of areas, ranging from marketing [1,2,3], entertainment [4,5], education [6,7,8], medicine [9,10], architecture and construction [11,12], manufacturing [13,14] to cultural heritage [15,16,17], one must understand the behavioural intention of end-users before embracing such novelty in a given context. Every time AR appears as a new approach to a specific context, predicting the behavioural intention of end-users to use it seems advantageous. Thus, it is essential to try to predict the users’ behavioural intention to use the technology through reliable tools.

This research deals with the possibility of using AR in Cultural Heritage (CH) sites for visitors, namely archaeological spaces. This premise takes into account that these sites are located outdoors and possess limited access to suitable technological solutions to explore them adequately. Furthermore, such places must remain naturally appealing for those who do not wish to be overwhelmed by technology. Previous acceptance studies focused on AR applications have been taken into account in order to understand if any of these tools would be suitable to evaluate the intention to use AR in such sites. According to the previous analysis, no acceptance model focused on understanding the intention to use AR in archaeological sites from the end-user’s perspective was identified. The literature review on technology acceptance models which are already verified and available for generalization among different areas revealed that a common approach is to adapt existing models to make them more suitable for a given technology or context.

A previous study [18] aimed to identify an existing and suitable model demonstrated that the Unified Theory of Acceptance and Use of Technology model (UTAUT) has the potential to understand the individuals’ behavioural intention to use AR technology in archaeological sites while presenting significant correlation between independent variables and the dependent variable of behavioural intention. That study, nevertheless, did not analyse the answers regarding the specific use of this technology from actual visitors of cultural heritage (CH) sites. In that study, online questionnaires were considered to be a suitable instrument to validate the acceptance model, but as stated, that model should be supplemented with more variables. This new proposal solves that problem by adding constructs to better understand end-users of AR technology when visiting a CH site. Ecological validity has been ensured in the current study by collecting results from actual visitors that were visiting archaeological sites.

Researchers of individual behaviour in different contexts have proposed a considerable diversity of models and theories in order to understand user behaviour in technology—which will enable to predict the performance of any voluntary act [19,20]. Furthermore, they have also been improving these proposals by combining them and, therefore, formulating new models to find more adequate solutions for each area of activity.

Considering the specificities of AR technology when compared with fully virtual environments, given its interaction and coexistence with the real world, and following the aforementioned lines of research, we propose a new acceptance model targeted to ascertain the intention to use augmented reality technology that could be generalized to different areas of implementation. This tool, the Augmented Reality Acceptance Model (ARAM), relies on the UTAUT model [21] to define some of its constructs, namely performance expectancy, effort expectancy, social influence, and facilitating conditions, and to understand how these variables relate to the behavioural intention to use the technology. In addition to UTAUT constructs, this model integrates the Mehrabian and Russel theory [22], taking into account a more recent review [23], in order to observe emotional states of individuals towards AR technology (hedonic motivation), computer anxiety feelings and trust issues (trust expectancy), a concept based on Chen’s theory [24] and on Kim’s et al. [25] approach. A new independent variable was also added to consider technological innovation.

Having been tested and validated outdoors by visitors from diverse archaeological sites in Portugal, this case study offers a flexible tool for accurately ascertaining the acceptance of using AR technology in CH.

## 2. Theoretical Background

The following literature review is divided into three main parts: (1) an overview of augmented technology is presented; (2) an overview of the most common models of evaluation of the acceptance and intention to use technology; (3) a range of previous studies regarding their choices taking into account available models in the literature.

### 2.1. Augmented Reality Overview

AR has seen a fast growth of technological advances and popularity across the world, becoming a frequent approach in different areas for different purposes. From medical aid to learning experiences at school and museums to pure entertainment, AR is an interesting and attractive way to innovate in the digital world. As Ronald T. Azuma sustained back in the nineties [26], AR can be considered a variant of VR with the particularity of blending the innovation of the digital world with the real surroundings. Several definitions of AR have been disseminated, including some variations of the technology as being Mixed Reality, or Extended Reality. This study does not intend to disclose the various definitions nor to compare the differences between them, whereas the definition of Azuma, widely adopted across the literature, stands for the current study. Therefore, when AR technology is referred to, it is implicit that it refers to any system that combines real and virtual content, as being an interaction in real-time, in the three-dimensional world, regardless of the technology used [26].

The technological development in the last decade brought new possibilities for AR to succeed as in the case of smartphones or head-mounted displays, such as HoloLens. Studies targeted for better understanding the impact of AR largely support its usage as it can be observed in systematics reviews focused on the advantages of AR in education [27], in medical training [28], in patients with autism [29], in industrial maintenance [30], in cultural heritage [31], among others. More generic studies related to the use of AR are also found, such as the systematic review of Dey et al. [32] or the survey of Billinghurst et al. [33]. When analysing previous AR studies and applications, it is frequently realised that those applications are mainly games or gamified applications of AR. This raises new questions related to the type of content and interactivity that is implemented, especially when covering AR solutions, since this technology can also vary in the number of virtual elements that are presented to the user—note the Virtuality Continuum concept presented by Milgram et al. [34].

### 2.2. Technology Acceptance Models in Literature

To comprehend the user’s intention to use technologies, a variety of models and theories have been developed to unravel this relation between users and technology. An early understanding related to the adoption of behaviours has been provided by the Theory of Reasoned Action [19], pointed out as a starting point for empirical and theoretical in the field of attitudes [20]. The authors stated that, by measuring behavioural intention, it is possible to predict the performance of any voluntary act. Largely used to predict behavioural intentions, it was based on this model that one of the most substantial and influential theories of human behaviour, the Technology Acceptance Model (TAM), was developed [35]. This model describes the motivational process mediating system characteristics and user behaviour, relating individual choices when adopting or not a technology when performing a task. For this analysis, measures related to the features of the system and capabilities are made to relate it to users’ motivation to use the system, which can affect their actual system use or non-use. A theoretical extension of TAM was presented as TAM 2 [36], which included additional theoretical constructs embracing social influence processes and cognitive instrumental processes. This acceptance model covers the evaluation of constructs such as perceived usefulness, perceived ease of use, intention to use, and actual usage behaviour. TAM 3 [37] results from the combination of TAM 2 with the model of the determinants of perceived use, creating new relationships, focused on interventions regarding potential pre- and post- implementations.

Despite the large number of studies conducted aiming to understand the factors that contribute to successful implementations of technology, DeLone and McLean looked at information system success as unachievable back then. Thus, they proposed the DeLone and McLean (D&M) Information Systems (IS) Success Model as a framework and model for measuring the complex-dependent variable in IS research, through six categories: system quality, information quality, use, user satisfaction, individual impact, and organisational impact [38]. This model was updated in 2003 attempting to capture the multidimensional and interdependent nature of IS success [39]. Service Quality was added and stated as an important dimension of IS success given the importance of IS support, especially in the proposed case study: e-commerce environment.

Consistent with DeLone and McLean’s proposal in 1992, a model called the Technology-to-Performance Chain was proposed in 1995 [40]. This approach stresses the linkage between constructs, reflecting the impact of information technology on performance. The importance of a construct known as Task-Technology Fit (TTF) on performance impacts is highlighted. TTF models explicitly include task characteristics, as the examples proposed in the Technology-to-Performance Chained, implying the matching of capabilities of the technology with the demands of the task. A common addition to TTF models is individual abilities, such as computer literacy, where its perception can be negatively affected between task and technology [41].

Among other new approaches, which have blended several models and theories striving for proposing new and more suitable models to better understand the acceptance of technology, a relevant example of these combinations is found: the Unified Theory of Acceptance and Use of Technology (UTAUT), proposed by Venkatesh et al. in 2003 [21]. This proposal unified eight theories and models of individual acceptance, namely, the Theory of Reasoned Action (proposed in 1988, as referred above), the TAM (proposed in 1986 as described above), the Motivational Model (proposed in 1992), the Theory of Planned Behaviour (proposed in 1991), the Theory of Planned Behaviour (1995), the Model of PC Utilisation (proposed in 1977), the Innovation Diffusion Theory (1995), and the Social Cognitive Theory (proposed in 1986). In their approach, they pointed out four constructs registered as significant to determine the behaviour intention of individuals to use technology, namely, performance expectancy, effort expectancy, social influence, and facilitating conditions. The UTAUT 2, presented in 2012 [42], provided three new constructs, namely, hedonic motivation, price value, and habit.

Observing the latest years and considering the relevant and useful acceptance models described so far, some researchers have been modifying a few relations between constructs or proposing some slight variations of these previous models by changing some constructs [43,44,45,46,47]. Those studies and others were analysed and certain results are pointed out onward during the discussion of this study, in Section 6.

### 2.3. Acceptance of Technology Case Studies

The applicability of acceptance models and theories has been a subject of study to accomplish a more accurate evaluation related to the degree of acceptance and, hence the use of technology in diverse acting areas.

Aiming to understand which models have been used lately for CH, brief research revealed TAM and UTAUT as the most common models used. For instance, Usoro et al. combined TAM and TTF to explore the user acceptance and use of e-commerce websites in tourism [48]. The UTAUT 2 model was used to understand online purchase intentions and actual online purchases [49]. The usage of AR for education was apprehended using the TAM model [50]. The users’ acceptance and use of the AR mobile application in Meleka—tourism sector—was evaluated using the UTAUT model [51]. The behavioural intention to use virtual reality in the learning process was evaluated by proposing the UTAUT model [44]. A study for acceptance of AR application within the urban heritage tourism context in Dublin proposed the use of TAM [47].

The acceptance of each technology may need specific requirements for its study. Therefore, aiming to provide a tool for ascertaining acceptance of AR technology by end-users in CH, a focused study regarding the acceptance of augmented reality in heritage contexts was conducted (from 2012 up to 2020) and, the list of found results, hitherto, is not very extensive. Table 1 presents some of the acceptance studies accomplished as well as the acceptance model used, and the sample size is specified. Questionnaires were the evaluation instrument used in all shown studies. An exception was found in one study [47], which used one-to-one interviews as an evaluation instrument.

As can be seen in Table 1, the TAM model was the most commonly used by researchers. UTAUT (or UTAUT 2) and DeLone & McLean are also frequently used. Sample sizes, when present, are between 31 and 241 participants.

The validation conducted by Haugstvedt et al. [52], where 200 answers were collected via the web and 42 as a street survey, highlighted their limitations in using different usage-modes and a different format of the scale in the web survey. For this reason, only the web survey was used to probe the TAM model. Furthermore, based on TAM and targeting online purchasing behaviour, Huang et al. [53] successfully added four dimensions from Holbrook’s study to the TAM research model. Results collected from young undergraduate and graduate students demonstrated a sustainable relationship between behaviour and the five key factors proposed in their study.

The UTAUT model was confirmed as a valuable tool to understand AR acceptance by Rodrigues et al. [57] and also by Marto et al. [18] but, the last raised the need to add more variables in order to better understand the way that acceptance and intention to use new technology are usually presented in other models and/or theories. Nonetheless, both studies affirm that their results, using the UTAUT model, allowed them to ascertain the potential to develop and apply their technologies in real contexts.

The literature review demonstrated that several studies aimed to adapt existing acceptance models to the idiosyncrasies of AR technology, such as the technology readiness by [55] or the cognitive innovation proposed by Huang et al. [53], which added constructs to the TAM model. However, as the authors acknowledge, a larger sample is needed for validation to allow further researchers to generalize results.

The role of AR—AR attitude and AR technology perception—on the intentional act was comprehensively discussed in previous literature [55], revealing great potential in tourism. This study proposed a useful tool from the heritage sites’ point of view as far as increasing their number of visitors was concerned. However, it did not look at the acceptance of AR technology in cultural heritage sites from the visitors’ perspective, i.e., how these would react to the use of AR technology during their visit. Doubts were also raised due to the difficulties tourists displayed when dealing with the technology. In addition, the applicability of this study is limited because it was conducted in a single heritage site and cannot, therefore, be generalized to others. Nonetheless, it becomes clear that the validation of AR acceptance models with end-users still requires clarification and justifies additional research for further implementation of AR technologies targeted at the general public. This becomes even more obvious if one takes into account the fact that, in order to have up-to-date knowledge of new technologies, it is crucial to continuously hold acceptance studies [18].

## 3. Methodology

This section addresses the baseline for the ARAM proposal, the methodology used for collecting data and the description of analysis procedures.

### 3.1. Baseline for Starting the Proposal

As the main goal of this study was to look for a prediction of behaviour concerning new technology, the proposal of the acceptance model was envisioned to be applied even before a concrete solution was available. Taking into account cases in which participants have no access to a prototype as a basis to propose a tool that can then be generalised, it was considered that developing a new model based on acceptance models which have questions targeted for after-experience feelings would be ineffective.

The unified theory of acceptance and use of technology models (UTAUT and UTAUT2) and its constructs related to expected behaviours towards a technological system seemed to better fit all purposes—when a technological solution is available for testing or not. The new constructs proposed for UTAUT 2, Price Value and Habit appeared to be misplaced because the current case study is not focused on commerce. In relation to the hedonic motivation construct, following the importance raised in the literature of hedonic-motivation on the adoption of a system [58], the ARAM model proposes a more detailed study on hedonic motivation than the approach presented in the UTAUT 2. This construct is further described in Section 4.1.

As mentioned in the literature review, the existing models, even though reliable for a series of purposes, give rise to some degree of uncertainty when aiming to predict AR acceptance. Thus, based on the UTAUT model, new constructs were added, relationships between them were adjusted and a new model was put to the test.

Therefore, the constructs are shortly listed as follows. A more detailed explanation is provided in Section 4.1. The constructs from the UTAUT model which were evaluated in the current study are performance expectancy, effort expectancy, social influence, facilitating conditions, and behavioural intention. The added constructs to the referred model are computer anxiety, hedonic motivation, trust expectancy, and technological innovation.

Three or four questions—described in Section 4.1—were designed for each construct. For each question there were several items which had to be classified according to a 7-point Likert scale, designed to characterize the level of agreement, ranging from “strongly disagree”, to “strongly agree”.

### 3.2. Collection Data

The questionnaires were physically distributed in several Portuguese archaeological sites as well as through online platforms and were made available in English and Portuguese.

#### 3.2.1. Paper Questionnaires

Under the consultation of the archaeologist of the Monographic Museum of Conimbriga-National Museum, Portugal, all Roman archaeological sites in Portugal were contacted to participate in this study in order to collect results from actual visitors of those sites. From a list of twelve sites, ten agreed to have their visitors questioned about their acceptance of using AR technology within their spaces.

Thus, aper questionnaires were then collected in the following Portuguese archaeological sites:

- “Citânia de Briteiros”, Martins Sarmento Society, Guimarães;
- “Tongóbriga”, Archaeological Site of Freixo, Marco de Canaveses;
- “Citânia de Sanfins”, Sanfins Archeological Museum, Paços Ferreira;
- “Monte Mozinho”, Penafiel Municipal Museum;
- “Conimbriga”, Monographic Museum of Conimbriga—National Museum, Condeixa-a-Velha;
- “Roman Villa of Rabaçal” (Penela’s Town Hall);
- “São Miguel de Odrinhas” (Sintra’s Town hall);
- “Tróia Roman Ruins” (Tróia Resort);
- “Miróbriga” (Alentejo’s Regional Culture Directorate);
- “São Cucufate” (Alentejo’s Regional Culture Directorate).

Contacts with these CH sites were initially conducted via email by the lead researcher, in order to request permission to include them in the acceptance study. When this strategy did not succeed even after sending a second email—which happened with most of the sites—direct phone calls were established. In several cases, the researcher had to personally travel to these sites and request the necessary permission for the study, on account of the various difficulties when contacting the person in charge. Once a request had been approved for a given site, questionnaires were either sent by post or delivered by hand, depending on the preferences in each case. In most situations, the researcher had to collect questionnaires in person once they had been filled since only a small number of entities agreed to send them by post.

The period for collecting these answers was planned to occur during the summer because that is when these archaeological sites are expected to have more visitors. Accordingly, results for this acceptance research were intended to be collected during the summer of 2018—from July to September. The way questionnaires were made available to visitors was agreed upon with the head of each archaeological site. In some cases, the researcher personally handed out the questionnaires and invited people to participate, while in other cases questionnaires were made available at the reception desk of the site and visitors participated voluntarily.

#### 3.2.2. Online Questionnaires

During the same period, online questionnaires were also distributed. Online platforms were used to publicize them, namely emails to the academic community at the School of Technology and Management of the Polytechnic of Leiria, newsletters from archaeological sites, and social network platforms such as YouTube, Facebook, and LinkedIn. To better contextualize this study for online participants, a promotion video was created and it can be viewed at the following link: https://youtu.be/u9i10xmwEaU, accessed on 28 December 2022 [59].

### 3.3. Analysis Procedures

Choosing a proficient factorial model is fundamental in Confirmatory Factorial Analysis (CFA). It is also indispensable to observe factorial loads and errors that statistically validate it and corroborate its adequacy to a specific study [60].

The analysis of the research model proposed in this study resorted to confirmatory factor analysis (CFA), using a Structural Equation Model (SEM) and SPSS/AMOS 27 software [61]. The mediation model was tested for validity and reliability of the measures following the literature and several research hypotheses were tested to determine the meaning of loadings and coefficients of each path [62].

The reliability of items and factors was verified through the calculation of Cronbach’s Alpha (α). This statistical technique is widely used and cited by several authors to demonstrate that the tests and scales that are built or adopted are relevant in explaining the results of research [63].

## 4. Research Model and Hypotheses

**Figure 1:**
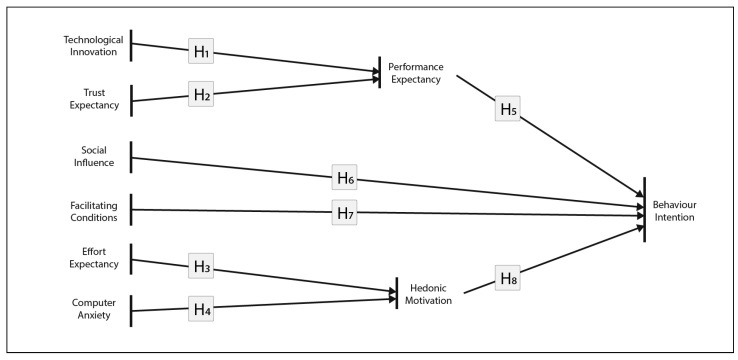
Proposed relationships between constructs before ARAM validation. The final version of ARAM can be found in Figure 2.

Aiming to create a tool for understanding individuals’ acceptance of augmented reality technology in heritage sites, we propose ARAM, illustrated in Figure 1. By measuring the behavioural intention of individuals, the proposed model was developed in order to understand the Behavioural Intention of individuals to use AR when visiting an archaeological site. In general, this model considers the following constructs to ascertain Behavioural Intention:

- Performance expectancy (PE) as the degree to which an individual believes he/she can benefit from using the technological system;
- Effort expectancy (EE) as the degree of ease associated with the use of the system;
- Social influence (SI) as the degree to which a person considers that everyone who is important to him/her believes that the new system should be used by each individual;
- Facilitating conditions (FC) as the degree to which the individual believes that he or she has the necessary conditions to access the technology;
- Computer anxiety (CA) as the degree of apprehension of an individual when facing the possibility of using a new technological system;
- Hedonic motivation (HM) as the degree of pleasure, arousal and dominance that an individual experiences when interacting with new technology;
- Trust expectancy (TE) as the degree to which a person trusts the information that he/she perceives while using the technological system;
- Technological innovation (TI) as the degree of desirable innovative features to be introduced in the technological system.

In this section, we present and explain the constructs and the hypotheses that were raised.

### 4.1. Conceptual Model

Deriving from the UTAUT model, the ARAM tool studies Behavioural Intention by analysing performance expectancy, effort expectancy, social influence, and facilitating conditions. In addition to these, computer anxiety, hedonic motivation, trust expectancy, and technological innovation are added. The traced relationships between these constructs and their impact on Behavioural Intention to use the AR system, as illustrated in Figure 1, are listed below. These hypotheses are comprehensively described in Section 4.2.

**H1_0_:**  *TI will influence the PE;*

**H2_0_:**  *TE will influence the PE;*

**H3_0_:**  *EE will influence the HM;*

**H4_0_:**  *CA will influence the HM;*

**H5_0_:**  *PE will influence the BI;*

**H6_0_:**  *SI will influence the BI;*

**H7_0_:**  *FC will influence the BI;*

**H8_0_:**  *HM will influence the BI.*

#### 4.1.1. Constructs from the UTAUT Model

This subsection briefly presents the definition of the constructs from the UTAUT model [21] and describes the items used for ascertaining each construct, while establishing their roles in the ARAM proposal.

#### Performance Expectancy

Venkatesh et al. [21] defined performance expectancy as the degree to which a person believes that using the system will help each individual to receive some sort of gain. In the original model, these gains were related to job performance. Aiming to propose a suited PE evaluation of the use of AR when visiting an archaeological site, the items used to evaluate PE were updated for the ARAM proposal as follows: PE 1. Information Quantity; PE 2. Speed in acquiring information; PE 3. Interest Enhancement; and PE 4. Unique approach to explore.

This construct embodies cultural enrichment, knowledge acquired from children, the capability to express information, gained cultural significance and the ability to recall information after more extended periods.

In line with the literature, PE is proposed to influence directly BI (H5) [42,43,44,45,46,64,65].

#### Effort Expectancy

The same authors defined effort expectancy as the degree of ease associated with the use of the system [21]. Aiming to propose a suited EE evaluation regarding the use of AR when visiting an archaeological site, the items used to evaluate EE were updated for the ARAM proposal as follows: EE 1. Ease of Use; EE 2. Clearness of Interaction; and EE 3. Ease to become skilful.

This construct includes the ease to learn how to use the technology, the fear to use AR in the context, the flexibility while interacting with the system and the reaction speed of interaction.

Although literature sustains the negative effect of EE on BI [42,44], this relation between both constructs was not statistically validated in several recent studies [43,45,64,66]. For this reason, effort expectancy is not proposed in ARAM as having a direct link with behavioural intention (H3), as the UTAUT model sustains.

#### Social Influence

Social influence was defined as the degree to which a person perceives that others believe each individual should use the new system [21]. Aiming to propose a proper SI evaluation regarding the use of AR when visiting an archaeological site, the items used to evaluate SI were updated for the ARAM proposal as follows: SI 1. Opinion of friends and family; SI 2. Influence of friends and family; and SI 3. Influence of people around.

Overall, in social influence, items are affected by significant human relationships and social status.

The perception of the SI role in acceptance studies is not unanimous according to the literature. Several studies sustain its direct link to BI [36,37,42,44,67], whereas others do not manage to validate that influence [43,44,45,64,65,68]. Other approaches even omit it [46]. Given this disparity, a relation between SI and BI is proposed for analysis in the case of the ARAM proposal (H6).

#### Facilitating Conditions

Venkatesh et al. [21] defined facilitating conditions as the degree to which an individual believes that an organizational and technical infrastructure exists to support the use of the system. The FC evaluation proposed in the UTAUT model seemed to fit the ARAM proposal. Thus, the items used to evaluate FC for the ARAM are the following: FC 1. Adequacy of resources; FC 2. Adequacy of knowledge; FC 3. Compatibility with other technologies; and FC 4. Adequacy of help available.

Thus, facilitating conditions include the sense of opportunity of AR technology in the archaeological space, technical issues, incidents and complexity of the content presented through the technology.

The influence of FC in the ARAM study was defined according to the literature by establishing a direct relationship with BI (H7) [42,44].

#### Behavioural Intention

According to Venkatesh et al. [21], it is expected that Behavioural Intention will have a significant positive influence on technology usage. Aiming to propose a suited BI evaluation regarding the use of AR when visiting an archaeological site, the items used to evaluate BI were slightly updated for the ARAM proposal as follows: BI 1. Intention to use as soon as possible; BI 2. Intention to use in the future; and BI 3. Intention to use regularly.

#### 4.1.2. Added Constructs for the ARAM

As mentioned above, new constructs were considered in addition to the reviewed UTAUT constructs, which are now described and substantiated. These constructs were added as a consequence of analysing previous studies that aimed to complete their acceptance models. The significance of these additions appeared to be relevant for the acceptance of AR technology since common points were spotted. Thus, the following subsections provide the motivations for adding these to the ARAM proposal.

#### Computer Anxiety

Technological developments and their impact on peoples’ feelings when using them have been a research matter for a long time. Individual avoidance and resistance towards new technologies have been related to anxiety, cognitive willingness and affective components. In the early ages of human–computer interaction, computer anxiety was defined as the individual’s apprehension or even fear when facing the possibility of using a computer [69], and later as the uneasiness or apprehension towards computers [70]. A Computer Anxiety Rating Scale (CARS) including 19 items was proposed [71]. Among them, feelings related to insecurity, apprehension or fear of making mistakes can be found. Computer anxiety is, therefore, essential to understanding how feelings of apprehension and fear towards a technological device can impact individual behaviour as far as using an AR system is concerned. Thus, the aforementioned definition was updated for this proposal in order to define computer anxiety as the degree to which an individual experiences apprehension or even fear when facing the possibility of iterating with technology.

The items proposed to evaluate CA for the ARAM are the following: CA 1. Nervousness; CA 2. Insecurity; and CA 3. Fearfulness.

Computer anxiety is also known in acceptance studies as a determinant of Perceived Ease of Use [72] and is frequently related to self-efficacy [73] and self-beliefs [74]. This construct has also been identified as strongly influencing technology usage [70,75]. Thus, aiming for a deeper understanding of this construct as an emotional state and taking into account that known results did not verify any direct relation between CA and BI [21], ARAM proposes the establishment of a relationship between computer anxiety and hedonic motivation (H4).

#### Hedonic Motivation

The emotion of an individual when using a system is determinant in technology acceptance [60]. Mehrabian and Russel categorized emotional responses into three basic dimensions: Pleasure, Arousal and Dominance [22]: Pleasure was classified as ranging from extreme pain or unhappiness to extreme happiness; Arousal was characterized as varying from sleep to frantic excitement; and Dominance was related to feelings of control, embracing restricted feelings in individual behaviour.

However, recent studies frequently present a two-dimensional model analysing pleasure and arousal to characterize the positive emotions towards a technological system. The following well-known examples should be taken into consideration: hedonic motivation characterized as fun and pleasure [42]; Joy being studied according to enjoyability, fun, boredom, annoyance, pleasure and unsatisfaction [58]; Hedonism defined as entertaining and pleasure-providing [76]. In these cases, hedonic motivation is found to be defined as fun or pleasure derived from using technology [42].

Bakker et al. [23] highlighted and demonstrated the importance of adding the third dimension of “dominance” to a complete range of human responses to ensure the distinction between feeling, thinking and acting. A connection between pleasure and affect, arousal and cognition, dominance and behaviour (connotation) was created, highlighting the similarities between the three response dimensions of Mehrabian and Russel and the ABC Model of Attitudes (ABC stands for Affect, Behaviour, and Cognition) [23]. Following these insights, the inclusion of dominance seems to bring more content to better fit the definition of hedonic motivation as the willingness of an individual to have positive experiences and avoid negative ones. Thus, this proposal perceives hedonic motivation as the degree to which an individual seeks pleasure and avoids pain while using technology.

Based on the briefly summarised literature review, the items proposed to evaluate hedonic motivation for the ARAM are the following: HM 1. Fun; HM 2. Arousal; and HM 3. Dominance.

The importance of this construct has been highlighted as well as its role in influencing the use of technological systems indirectly [70,72,74,75]. It is uncertain whether it has any direct influence on BI [21,77], sometimes even being referred to as not influencing behaviour [66]. Aiming to better understand HM´s role in BI, ARAM proposes a direct relation between HM and BI (H8).

#### Technological Innovation

Innovativeness was added to an acceptance model, this study includes the construct of technological innovation. Personal Innovativeness is defined in the literature as the ability to adopt information technology innovations earlier than others and it is related to individual traits of technology adoption [78]. The technological innovation construct, however, is not intended to study any personal traits in RAM; it aims to understand how novelty can be welcomed by the user while using a specific technology. Similarly to the items used to test Personal Innovativeness, in which questions are intended to assess the individual’s willingness to use technology, technological innovation considers new features in technology and presents them to users as a means to observe if those features are perceived positively when added to a technological experience. Hence, technological innovation is understood as the degree to which an individual wishes to have access to innovative features in the technological system.

The items used for construction can vary depending on the technology addressed by the study and they should cover specific features that can be provided by a specific technology. The items in ARAM are related to the addition of specific stimuli to the technological system to be implemented, in particular, the addition of sound, smell and temperature. Accordingly, the items used for the current study are as follows: TI 1. Audio stimulus; TI 2. Olfactory stimulus; and TI 3. Haptic stimulus.

Aiming to ascertain the role of TI in the ARAM proposal, a relationship between TE and PE was defined (H1) as a means to encompass the novelty character of the technology and the innovation it brings when adding new features to the users’ experience.

#### Trust Expectancy

Trust in technology is frequently related to consumers’ trust and credibility when using a system from a commercial point of view [24,25,53,79]. A model representing the factors that contribute to consumer trust in an online travel site was developed, in order to understand consumer confidence in e-commerce [24]. The evaluation of web-based information perceived credibility was also carried out, highlighting different perceptions that web credibility may sustain [79]. Trust has ultimately been defined as belief, confidence, attitude towards or expectation about another party’s trustworthiness in electronic commerce [25].

By expanding this concept beyond the scope of commerce and taking the literature on ecological validity into consideration, which is perceived as the degree to which the consumer believes in the simulated environment [80], the construct of trust expectancy was broadened in the current research in order to include technological integrity, reliability and trustworthiness of the contents provided by the technological device. Hence, instead of focusing on e-commerce trust [24] or on how natural or realistic the virtual object may seem to the user [80], this dimension seeks to grasp how reliable and trustworthy the use of AR technology can be. Taking into account the purpose of this research, the concept of trust expectancy was also updated in order to correspond to the degree to which a person trusts in the contents that he or she perceives when interacting with the technological system.

Aiming to evaluate TE towards the use of AR when visiting an archaeological site, the following items were established: TE 1. Credibility; TE 2. Reliability; and TE 3. Trustworthiness.

A relation between TE and PE was defined (H2) to ascertain the role of TE in the ARAM proposal due to the importance that these feelings of confidence may have on users and their perception while experiencing a system.

### 4.2. Formulation of Hypotheses

The hypotheses for this study were firstly based on the unified theory presented by Venkatesh et al. [21] regarding PE, EE, SI, and FC to which new constructs, such as CA, HM, and TE were added. As a result of the research that was briefly described in Section 4.1.2, new constructs were also created, thus triggering new hypotheses and new relations between constructs.

**H1:**  *The stronger technological innovation is, the stronger performance expectancy will be.*

**H2:**  *The stronger trust expectancy is, the stronger performance expectancy will be.*

**H3:**  *The stronger effort expectancy is, the weaker hedonic motivation will be.*

**H4:**  *The stronger computer anxiety is, the weaker hedonic motivation will be.*

**H5:**  *The stronger performance expectancy is, the stronger Behavioural Intention to use the system will be.*

**H6:**  *The stronger social influence is, the stronger behavioural intention to use the system will be.*

**H7:**  *The stronger facilitating conditions are, the stronger the Behavioural Intention to use the system will be.*

**H8:**  *The stronger hedonic motivation is, the stronger behavioural intention to use the system will be.*

Table 2 summarises the hypotheses raised according to dependent and independent variables and establishes relationships between constructs.

## 5. Data Analysis and Results

The Confirmatory Factorial Analysis (CFA) was conducted according to what was described in the previous Section 3.3. The model was tested with the dimensions following the constructs presented in Section 4.1 represented as technological innovation (TI), trust expectancy (TE), social influence (SI), facilitating conditions (FC), effort expectancy (EE), computer anxiety (CA), performance expectancy (PE), hedonic motivation (HM) and behavioural intention (BI).

Regarding the reliability of items and factors, it was verified that there is a good total internal consistency (α = 0.883) for the 528 respondents sample. According to Cronbach’s Alpha (α), the level of internal consistency for all the items that make up the model is higher than 0.8, thus revealing validity and internal and explanatory reliability.

### 5.1. Confirmatory Factorial Analysis (CFA)

**Figure 2:**
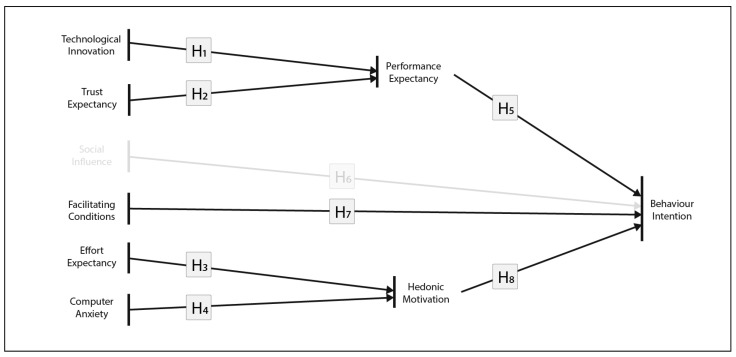
Final ARAM. The grey representation of the SI construct illustrates its drop from the model.

The model proposed in Section 4.1, which was analysed according to Section 3.3 did not support the H3 hypothesis. Therefore, the SI construct was eliminated from the initial model proposal. The final ARAM model is presented in Figure 2.

**Figure 3:**
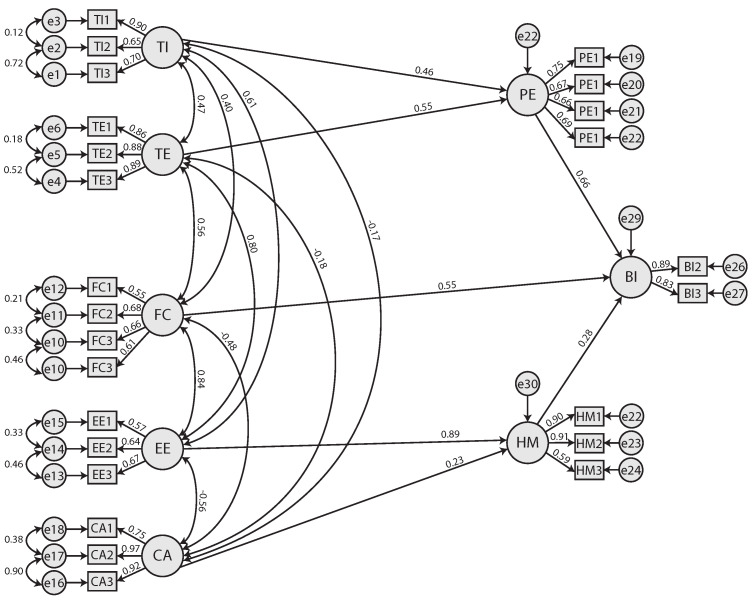
Final Research Model (Technological Innovation (TI), Trust Expectancy (TE), Facilitating Conditions (FC), Effort Expectancy (EE), Computer Anxiety (CA), Performance Expectancy (PE), Hedonic Motivation (HM) and Behavioural Intention (BI)).

After dropping the SI construct, the performed CFA demonstrated with a maximum probability that the tested model presented an adequate adjustment (*χ*^2^ = 1125.978, *p* = 0.001, df = 314, *χ*^2^/df = 3.586, RMSEA = 0.070, CFI = 0.919, GFI = 0.861) [60]. Figure 3 shows the standardized path coefficients in which all the paths of the model were significant (*p* < 0.001).

### 5.2. Validity and Reliability of the Measurement Model

The Average Variance Extracted (AVE), Composite Reliability (CR) and Cronbach alpha (α) were examined to assess the convergent validity and reliability of the model, only taking into account measurement items whose factor loadings (AVE > 0.5; CR > 0.7; α> 0.7) varied within acceptable statistical parameters [62]. As for the sample, it satisfies the criteria for structural equation analysis, which proposes a minimum of 5 observations for each variable of the model [81,82] suggest similar limits but propose complex models with few indicators per construct and larger samples. According to the sources previously referred to, it can be stated that the sample used is sufficient for the use of structural equation models. The structural equation model presented allows a multivariate analysis that can test more complex models than the traditional linear regression [83].

In Table 3, it is possible to see a summary of the hypotheses that were tested. Table 4 and Table 5 show the obtained regression weights and the standardized regression weights.

## 6. Discussion

In this section, we discuss the proposed ARAM model as an instrument to measure acceptance of the use of AR in the cultural heritage context.

### 6.1. Hypotheses Discussion

A brief discussion of each hypothesis that was raised, validated or not, is thereafter presented. To better contextualise this discussion, bore jumping into the constructs discussion, some brief insights are pointed out.

### 6.2. Relevant Considerations

In addition to the insights provided in this discussion, it is necessary to point out some objective gaps in the literature that were taken into consideration while conducting this research.

The necessity to include new constructs in addition to UTAUT was raised in a previous acceptance study based on the UTAUT model to ascertain the intention to use AR as a new technology in archaeological sites [18]. This necessity is directly met by this proposal of a new model based on UTAUT.

Taking into account some limitations identified in the literature, this study also addresses the need to extend an acceptance study to several heritage sites—therefore not being restricted to a single one while collecting results—[55], while being strongly supported by end-users and their point of view [52,53]. It also tackles the issue of reduced sample size [53,55] or limited samples targeted for a particular generation [66] by integrating a wide and diversified sample of participants, thus allowing to comfortably propose ARAM as an acceptance model for AR technology for a variety of scenarios.

In addition, ARAM establishes a role for computer anxiety in technology acceptance which is then combined with the diversity of the research sample, thus allowing an adequate approach to the concerns posed by the literature pertaining to tourists’ difficulties when dealing with the technology in question [55].

#### 6.2.1. Performance Expectancy Relationships

PE, in line with previous research [42,43,44,45,46,64,65], is demonstrated to influence BI in a way that confirms that the stronger performance expectancy is, the stronger behavioural intention to use the system will be (H5). In addition to this, the research shows that the PE of a novel technology is influenced by the innovations that are offered by this technology and the level of trust it achieves. It was observed that the stronger technological innovation is, the stronger performance expectancy becomes (H1). As far as can be concluded, this construct is a novelty in a technology acceptance model. It is possible to relate it with Personal Innovativeness and its role in the user’s intention to use a new technology [78] because both suggest innovativeness, i.e., a willingness to change [84]. In line with the conclusions of the literature that pointed to the Personal Innovativeness construct as having key relationships in technology acceptance, the TI construct in ARAM demonstrates its influence on a technology acceptance study.

Furthermore, it was observed that the stronger trust expectancy is, the stronger the performance expectancy will be (H2). Even though not commonly found in acceptance models, results are in line with the literature that verified Trust as having a positive effect on Loyalty [25]—Loyalty, in this study, is related to the perceived intention to act, such as to visit a website, to use online virtual communities or to purchase online. Thus, the validation of trust expectancy in the ARAM proposal demonstrates an indirect relation to BI, therefore acquiring a significant role in a technology acceptance model.

#### 6.2.2. Hedonic Motivation Relationships

Updating the hedonic motivation construct from previous acceptance studies that embraced arousal and fun [42,53,58,76], by adding the third dimension “control”, it was necessary to better understand people’s emotions and feelings [22,23]. Here, results demonstrate that the stronger the participants’ hedonic motivation regarding pleasure, fun and control on using the system is, the stronger the behavioural intention to use it will be (H8). It should be noted that these results are in line with previous research [42] but not with a more recent study [66], which presents hedonic motivation as not influencing behavioural intention to use. Even though both consider HM as a two-dimensional (fun and pleasure) approach, our study reinforces the importance of HM and its three dimensions on acceptance studies as necessary items to ascertain this construct.

Research also demonstrates HM to be negatively influenced by EE and by CA (H3 and H4). Aiming to discover new relationships for this construct in line with previous studies that relate EE to other constructs apart from BI [45,64], the ARAM proposal relates EE to hedonic motivation. Thus, this model advocates its negative effect on hedonic motivation, i.e., the stronger effort expectancy is, the weaker the user’s hedonic motivation will be (H3). In fact, effort expectancy has also been changing its role in previous studies as in the case of its positive effect on performance expectancy [64]. In other cases, it is simply not considered in the model [46].

A role and a scale for understanding CA when ascertaining the acceptance of technology were also proposed in this research, following the literature suggestions for understanding the role of computer anxiety in technology acceptance models [72]. According to the results we gathered, HM is influenced by computer anxiety in a way that can be best described as the stronger computer anxiety is, the weaker the hedonic motivation becomes (H4). This relation is roughly in line with previous studies that took into consideration the users’ CA regarding emotional states as in the case of the mediation of computer anxiety identified in behavioural and emotional dimensions [85]. In that sense, it provides another perspective on the question of its non-significance when directly related to BI [21,77] and its known influence in the use of technological systems [70,72,74,75]. Thus, the validity of this hypothesis in ARAM supports a significant role of computer anxiety on an acceptance model due to its indirect relation to the behaviour intention to use.

#### 6.2.3. Facilitating Conditions’ Impact on Behavioural Intention

In line with previous research [42,44], FC is demonstrated to influence BI as one of the direct determinants of the users’ behaviour. The stronger facilitating conditions are, the stronger the behavioural intention to use the system is (H7). Nonetheless, some studies identify FC as not being related to BI. Instead, a positive effect is found on effort expectancy [64], as predicting performance expectancy [46], or even as influencing the perceived ease of use and perceived usefulness [47]. Other studies do not include this construct, even though they use the UTAUT model [43,65], or do not validate it while being conducted [45].

#### 6.2.4. The Drop of Social Influence

Hypothesis H6, which stated that the stronger the social influence is the stronger the behavioural intention to use the system becomes, was not supported. The impact of SI’s dubiousness is in line with the differing results found in the literature and the discussion it has recently originated. Despite its significance in some research [36,37,42,44,67], it has been demonstrated not to be significant when predicting BI in several studies [43,44,45,64,65,68], to the point of even being omitted in some approaches [46]. Nonetheless, SI is still seen as having an important role when embracing new technology, not on a social but on a professional level [86], especially when dealing with mandatory technology acceptance—i.e., it is not significant when the technology use is optional [21]. SI was also statistically demonstrated to have a positive effect on PE [64].

In addition to the initial model proposals [36,77], SI was also shown to be significantly relevant in BI in later research [44].

A new opportunity for technological novelties was created by adding the construct of technological innovation. To generalize this construct for further applications, it should be noted that the three novelties added to a system should be related between them. The example provided was focused on sensorial innovation.

### 6.3. Limitations and Future Work

There are some limitations which should be pointed out. By observing the results for facilitating conditions across the various studies, it became obvious that the technological systems in question also differed and pointed to distinct types of facilitating conditions and issues depending on each case. Given the subjectivity of this construct, a better specification of facilitating conditions should be developed for further studies. It should be noted that the items used to ascertain the facilitating conditions in the conducted study—according to the UTAU model—do not objectively account for any specifications related to the technological device in use. Even though acknowledging that having a broader concept can make it more reliable for wider situations, if one should consider, for instance, a smartphone as the technological device to use an AR system, users may not regard issues such as battery life or hardware performance as facilitating conditions.

As for technological innovation, it becomes clear that deeper research can be carried out. Adding novelties to a given technology can be a wide and complex endeavour but a list of topics with available options for this construct should be created and properly validated as it would enable a simpler generalization of this construct.

Users’ cultural and individual backgrounds should also be elaborated on in future research to consolidate the model. This type of study frequently deals with heterogeneous groups of people—different ages, genders, cultures and backgrounds. As far as cultural studies are concerned as well as their view of cultural aspects as being influential, it is important to highlight the proper understanding of such factors because they do have a significant role. Thus, cultural moderators are frequently taken into consideration in studies related to the acceptance and use of technology (e.g., [77,87]). Others specifically aim to ascertain cultural differences, such as [88,89], in order to understand how different cultures react differently to the same proposals. Hence, the following stage of such research should aim to identify the moderators for each construct.

This model was validated through several archaeological sites and online platforms while introducing AR as the technology to use. Nonetheless, despite being very wide and diversified, the validation was developed for a CH context, where people are free to use the technology according to their will. Hence, even though ARAM may be applied to several situations, this research did not consider scenarios in which users could be pushed to use a technology to progress in their careers or to directly benefit from their use of the technology.

## 7. Conclusions

This study proposed and validated a technology acceptance model targeted for AR implementations by having as a starting point the well-known UTAUT model and by adding new constructs. The main goal was to better understand the behavioural intention to use AR technology in CH contexts.

The results obtained in ARAM analysis demonstrated that the behavioural intention to use AR can be ascertained by analysing users’ feelings regarding their performance expectancy, facilitating conditions, and hedonic motivation. In addition to these direct effects on BI, ARAM identified a significant influence of technological innovation and trust expectancy on performance expectancy. Similarly, but as a negative influence, effort expectancy and computer anxiety were identified as having a significant impact on hedonic motivation to use AR technology. This research also brought back the third dimension of dominance to complement pleasure and fun, aiming to characterize hedonic motivation.

ARAM added two motivational factors related to how the user feels towards technology—computer anxiety and hedonic motivation. The relation between CA and HM supports the importance of participants’ emotions when using a system because they interfere with their behavioural intention to use it.

With archaeological sites as the context to use AR technology, the proposed tool for understanding the behavioural intention to use AR was validated with participants that were visiting archaeological sites in Portugal and with possible future visitors of CH sites by having results collected through several online platforms.

As stated, AR’s popularity enables its implementation in a wide range of areas. Its success depends on very different factors but it is possible to increase its success by predicting the acceptance of the technology on the end-user side. The ARAM model presents a validated questionnaire to use as a tool to predict the behavioural intention to use AR in cultural heritage. The end-user validation and the diversity of the participants assure ecological validity and increase the chance of accurately ascertaining the user’s acceptance of AR to a wider set of areas, even if it is not cultural heritage scenario.

Thus, every time AR appears as a new solution for a specific context, we believe that ARAM, in which the questionnaire is available for usage in the Supplementary Materials section at the end of this manuscript, can be generalized and applied to other areas.

## Figures and Tables

**Table 1:** Previous acceptance of augmented reality technology studies found, from 2012 until now.

Context	Model	Sample	Reference
AR in Cultural Heritage	TAM	242	[52]
AR interactive technology to enable consumers to try on clothes online	TAM	220	[53]
AR in a natural park	D&M	241	[54]
AR for tourism: destinations and attractions	TAM	145	[55]
AR Travel Guide	UTAUT2	105	[56]
AR for education: helping engineering students to solve problems	TAM	122	[50]
Mobile AR app to show campus-related information on a map	UTAUT D&M	look	[46]
AR in urban heritage tourism	TAM	44	[47]
AR in Archaeological Sites	UTAUT	31	[18]
AR in Museums	UTAUT	31	[57]

**Table 2:** Summary of dependent and independent variables according to ARAM proposal and hypothetical scenarios created.

IV	DV	Hypothetical Scenario
TI	PE	Performance expectancy is positively influenced by technological innovation.
TE	PE	Performance expectancy is positively influenced by trust expectancy.
EE	HM	Hedonic motivation is negatively influenced by effort expectancy.
CA	HM	Hedonic motivation is negatively influenced by computer anxiety.
PE	BI	Behavioural intention is positively influenced by performance expectancy.
SI	BI	Behavioural intention is positively influenced by social influence.
FC	BI	Behavioural intention is positively influenced by facilitating conditions.
HM	BI	Behavioural intention is positively influenced by hedonic motivation.

**Table 3:** Research hypotheses and statistical results.

Hypothesis	Relation	Regression Coefficient	Standard Error	t	*p*-Value	Result
H1	TI → PE	0.250	0.032	7.704	<0.001	Supported
H2	TE → PE	0.431	0.039	11.043	<0.001	Supported
H3	EE → HM	0.863	0.079	12.917	<0.001	Supported
H4	CA → HM	0.208	0.033	3.694	<0.001	Supported
H5	PE → BI	0.302	0.097	4.625	<0.001	Supported
H6	FC → BI	−0.497	0.219	−2.444	<0.05	Supported
H7	HM → BI	0.192	0.090	2.786	<0.05	Supported

**Table 4:** Regression Weights: (Group number 1—Default model).

	Estimate	S.E.	C.R.	*p*
PE ← TI	0.285	0.030	9.384	***
PE ← TE	0.407	0.036	11.414	***
HM ← EE	1.051	0.081	12.918	***
HM ← CA	0.140	0.035	3.985	***
BI ← FC	0.065	0.057	1.140	0.254
BI ← PE	1.039	0.083	12.541	***
BI ← HM	0.355	0.065	5.420	***
TI3 ← TI	1.000			
TI2 ← TI	0.942	0.049	19.141	***
TI1 ← TI	0.977	0.069	14.084	***
TE3 ← TE	1.000			
TE2 ← TE	0.992	0.028	35.277	***
TE1 ← TE	0.941	0.040	23.260	***
FC3 ← FC	1.000			
FC2 ← FC	1.037	0.067	15.455	***
FC1 ← FC	0.742	0.065	11.468	***
EE3 ← EE	1.000			
EE2 ← EE	0.939	0.053	17.779	***
EE1 ← EE	0.927	0.079	11.762	***
CA3 ← CA	1.000			
CA2 ← CA	1.015	0.063	16.056	***
CA1 ← CA	0.749	0.070	10.725	***
PE1 ← PE	1.000			
PE2 ← PE	0.905	0.061	14.890	***
PE3 ← PE	1.136	0.077	14.696	***
HM1 ← HM	1.000			
HM2 ← HM	1.090	0.040	27.600	***
HM3 ← HM	0.894	0.060	14.960	***
BI2 ← BI	1.000			
BI3 ← BI	0.941	0.041	22.797	***
FC4 ← FC	0.835	0.077	10.872	***
PE4 ← PE	1.137	0.074	15.337	***

*** *p* < 0.001 (Level of significance *p* < 0.001).

**Table 5:** Standardized Regression Weights: (Group number 1—Default model).

	Estimate
PE ← TI	0.464
PE ← TE	0.548
HM ← EE	0.888
HM ← CA	0.234
BI ← FC	0.059
BI ← PE	0.663
BI ← HM	0.275
TI3 ← TI	0.700
TI2 ← TI	0.651
TI1 ← TI	0.898
TE3 ← TE	0.891
TE2 ← TE	0.876
TE1 ← TE	0.856
FC3 ← Fc	0.665
FC2 ← Fc	0.684
FC1 ← Fc	0.548
EE3 ← EE	0.666
EE2 ← EE	0.642
EE1 ← EE	0.571
CA3 ← CA	0.920
CA2 ← CA	0.971
CA1 ← CA	0.747
PE1 ← PE	0.755
PE2 ← PE	0.669
PE3 ← PE	0.661
HM1 ← HM	0.898
HM2 ← HM	0.909
HM3 ← HM	0.593
BI2 ← BI	0.888
BI3 ← BI	0.826
FC4 ← FC	0.612
PE4 ← PE	0.688

## Data Availability

Data available on request.

## Supplementary Materials

The following supporting information can be downloaded at: https://www.mdpi.com/article/10.3390/jimaging9030073/s1, ARAM Questionnaire.

## Abbreviations

The following abbreviations are used in this manuscript:

AR	Augmented Reality
ARAM	Augmented Reality Acceptance Model
AVE	Average Variance Extracted
BI	Behavioural Intention
CA	Computer Anxiety
CARS	Computer Anxiety Rating Scale
CFA	Confirmatory Factorial Analysis
CH	Cultural Heritage
CR	Composite Reliability
D&M	DeLone and McLean
EE	Effort Expectancy
FC	Facilitating Conditions
HM	Hedonic Motivation
IS	Information Systems
PE	Performance Expectancy
SEM	Structural Equation Model
SI	Social Influence
TAM	Technology Acceptance Model
TE	Trust Expectancy
TI	Technological Innovation
TTF	Task-Technology Fit
UTAUT	Unified Theory of Acceptance and Use of Technology
