# Supplementary material for: ARAM: A Technology Acceptance Model to Ascertain the Behavioural Intention to Use Augmented Reality
Source: J Imaging. 2023 Mar 21;9(3):73. doi: 10.3390/jimaging9030073 (PMC10053472; doi:10.3390/jimaging9030073)
Supplement: Supplementary file 1 [file jimaging-09-00073-s001.zip › jimaging-2160861-supplementary.pdf]

ARAM Questionnaire

		Strongly Disagree ← … 1	2	3	4	5	6	… → Strongly Agree 7
[1]	Using augmented reality may help me get more information about the archaeological space.	◯	◯	◯	◯	◯	◯	◯
[2]	Using augmented reality may help me get information about the archaeological space more quickly.	◯	◯	◯	◯	◯	◯	◯
[3]	Using augmented reality may increase my interest in archaeological spaces.	◯	◯	◯	◯	◯	◯	◯

		Strongly Disagree ← … 1	2	3	4	5	6	… → Strongly Agree 7
[4]	I think that augmented reality is easy to use.	◯	◯	◯	◯	◯	◯	◯
[5]	I think that my interaction with augmented reality will be clear and understandable.	◯	◯	◯	◯	◯	◯	◯
[6]	It will be easy for me to become skilful at using augmented reality.	◯	◯	◯	◯	◯	◯	◯

		Strongly Disagree ← … 1	2	3	4	5	6	… → Strongly Agree 7
[10]	I have the resources necessary to use augmented reality (e.g. smartphone).	◯	◯	◯	◯	◯	◯	◯
[11]	I have the knowledge necessary to use augmented reality.	◯	◯	◯	◯	◯	◯	◯
[12]	Augmented reality is compatible with other technologies I use.	◯	◯	◯	◯	◯	◯	◯
[13]	I can get help from others if I have difficulties using augmented reality.	◯	◯	◯	◯	◯	◯	◯

		Strongly Disagree ← … 1	2	3	4	5	6	… → Strongly Agree 7
[14]	Using augmented reality in archaeological spaces can be fun.	◯	◯	◯	◯	◯	◯	◯
[15]	Using augmented reality in archaeological spaces can be exciting.	◯	◯	◯	◯	◯	◯	◯
[16]	I think that I will feel like I am in control of the augmented reality experience.	◯	◯	◯	◯	◯	◯	◯

		Strongly Disagree ← … 1	2	3	4	5	6	Strongly Agree … → 7
[17]	I feel nervous about using augmented reality.	○	○	○	○	○	○	○
[18]	I feel insecure about my ability to use augmented reality.	○	○	○	○	○	○	○
[19]	I'm afraid to use augmented reality for fear of making mistakes that I cannot correct.	○	○	○	○	○	○	○

		Strongly Disagree ← … 1	2	3	4	5	6	Strongly Agree … → 7
[20]	Augmented reality applied to archaeological spaces may be credible.	○	○	○	○	○	○	○
[21]	Augmented reality applied to archaeological spaces may be reliable.	○	○	○	○	○	○	○
[22]	Augmented reality applied to archaeological spaces may be trustworthy.	○	○	○	○	○	○	○

		Strongly Disagree ← … 1	2	3	4	5	6	Strongly Agree … → 7
[23]	In addition to seeing virtual images with augmented reality, I would like to listen to sounds related to the archaeological space.	○	○	○	○	○	○	○
[24]	In addition to seeing virtual images with augmented reality, I would like to smell aromas related to the archaeological space.	○	○	○	○	○	○	○
[25]	In addition to seeing virtual images with augmented reality, I would like to feel sensations (e.g. temperature) related to the archaeological space.	○	○	○	○	○	○	○
[26]	With augmented reality I will be able to explore archaeological spaces in a way that wouldn't be possible without it.	○	○	○	○	○	○	○

		Strongly Disagree ← ... 1	2	3	4	5	6	Strongly Agree ... → 7
[27]	I would like to use augmented reality in archaeological spaces as soon as possible.	○	○	○	○	○	○	○
[28]	I plan to use augmented reality applied to archaeological sites in the future.	○	○	○	○	○	○	○
[29]	I will always try to use augmented reality when visiting archaeological sites.	○	○	○	○	○	○	○

		Very Bad ← ... 1	2	3	4	5	6	Very Good ... → 7
[30]	I classify my level of knowledge related to archaeology as:	○	○	○	○	○	○	○
[31]	I classify my level of knowledge related to how to use augmented reality as:	○	○	○	○	○	○	○
[32]	I classify my level of reading of English to understand this questionnaire as:	○	○	○	○	○	○	○
